# Suppression of spin-exciton state in hole overdoped iron-based superconductors

**DOI:** 10.1038/srep23424

**Published:** 2016-03-23

**Authors:** C. H. Lee, K. Kihou, J. T. Park, K. Horigane, K. Fujita, F. Waßer, N. Qureshi, Y. Sidis, J. Akimitsu, M. Braden

**Affiliations:** 1 National Institute of Advanced Industrial Science and Technology (AIST), Tsukuba, Ibaraki 305-8568, Japan; 2 Heinz Maier-Leibnitz Zentrum (MLZ), Technische Universität München, D-85748 Garching, Germany; 3 Aoyama Gakuin University, Sagamihara 252-5258, Japan; 4 II. Physikalisches Institut, Universität zu Köln,50937 Cologne, Germany; 5 Laboratoire Léon Brillouin (LLB), C.E.A./C.N.R.S., F-91191 Gif-sur-Yvette Cedex, France

## Abstract

The mechanism of Cooper pair formation in iron-based superconductors remains a controversial topic. The main question is whether spin or orbital fluctuations are responsible for the pairing mechanism. To solve this problem, a crucial clue can be obtained by examining the remarkable enhancement of magnetic neutron scattering signals appearing in a superconducting phase. The enhancement is called spin resonance for a spin fluctuation model, in which their energy is restricted below twice the superconducting gap value (2Δ_s_), whereas larger energies are possible in other models such as an orbital fluctuation model. Here we report the doping dependence of low-energy magnetic excitation spectra in Ba_1−x_K_x_Fe_2_As_2_ for 0.5 < x < 0.84 studied by inelastic neutron scattering. We find that the behavior of the spin resonance dramatically changes from optimum to overdoped regions. Strong resonance peaks are observed clearly below 2Δ_s_ in the optimum doping region, while they are absent in the overdoped region. Instead, there is a transfer of spectral weight from energies below 2Δ_s_ to higher energies, peaking at values of 3Δ_s_ for x = 0.84. These results suggest a reduced impact of magnetism on Cooper pair formation in the overdoped region.

One of the most plausible mechanisms for Cooper pair formation in iron-based superconductors is spin-mediated superconductivity. In this model, the superconducting gap symmetry is predicted to be s_±_ with opposite phases between electron and hole Fermi surfaces^1^,^2^. Another possibility is superconductivity induced by orbital fluctuations, where the gap symmetry of s_++_ is expected with identical phases between electron and hole Fermi surfaces^3^,^4^. The orbital fluctuation model has been experimentally supported by ultrasound measurements^5^, impurity effects of superconducting transition temperature (*T*_*c*_)^6^,^7^ and Raman scattering^8^. To judge which model is appropriate, further studies are required.

Direct insight into determining a superconducting mechanism can be obtained by the examination of spin resonance. In the spin fluctuation model, the resonance mode is interpreted as a bound spin-exciton state (particle-hole excitation)^1^,^2^,^9^,^10^; therefore, its energy (*E*_*res*_) must stay below 2Δ_s_. In contrast, in the orbital fluctuation model, there is an enhancement of scattering at energies exceeding 2Δ_s_^11^, and scattering at low energies is suppressed in the superconducting state. This can be explained by the suppression of particle-hole scattering at low energies due to the opening of the superconducting gap. Several inelastic neutron scattering (INS) experiments have previously been performed on spin resonance modes of FeAs-based superconductors, especially in electron-doped BaFe_2_As_2_^12^,^13^,^14^,^15^,^16^,^17^,^18^,^19^,^20^,^21^,^22^,^23^. It has been found that the resonance correlates strongly with magnetism, supporting the spin-exciton model^13^,^14^,^15^,^16^. In contrast, for hole-doped BaFe_2_As_2_, doping dependence remains unclear in spite of higher *T*_*c*_^18^,^19^,^20^,^21^,^22^.

Thermal conductivity measurements on Ba_1−x_K_x_Fe_2_As_2_ suggest that the superconducting gap symmetry is transformed from s to d-wave with doping in a heavily overdoped region^24^. On the other hand, angle-resolved photoemission (ARPES) measurements demonstrate that the full s-wave gap varies to a nodal s-wave gap above x = 0.7^25^,^26^. Although the gap symmetry in the heavily hole overdoped region remains controversial, there is a consensus that a superconducting gap structure changes dramatically with doping. To clarify how the relationship between magnetism and superconductivity varies with changing the gap structure, we studied the doping dependence of spin fluctuations as well as that of spin resonances in Ba_1−x_K_x_Fe_2_As_2_ using the INS technique on single-crystalline samples.

## Results

**Figure 1:**
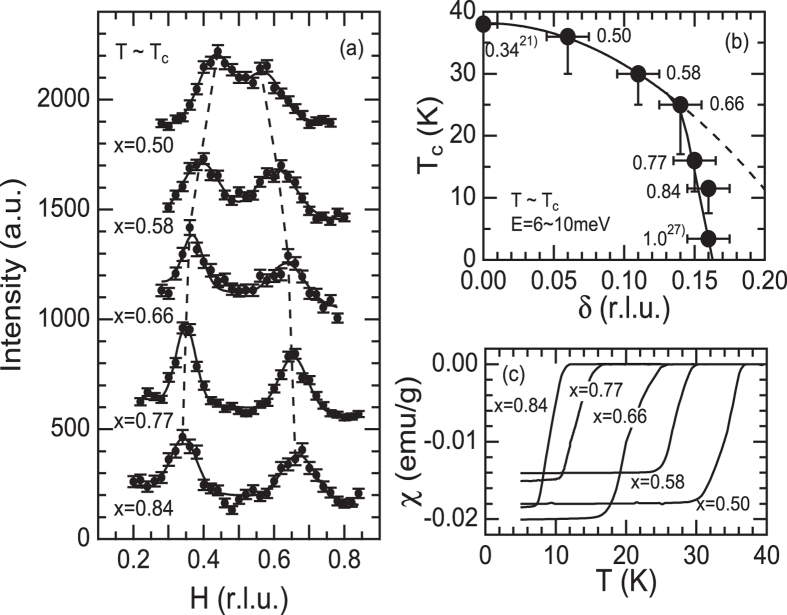
(**a**) Q-spectra of Ba_1−x_K_x_Fe_2_As_2_ along (*H, H*, 1) in the normal state. Energies and temperatures are E = 8 meV, T = 42 K for x = 0.50; E = 8 meV, T = 36 K for x = 0.58; E = 8 meV, T = 36K for x = 0.66; E = 8 meV, T = 22 K for x = 0.77 and E = 6 meV, T = 15 K for x = 0.84. (**b**) T_c_ vs. incommensurability δ in the normal state close to T_c_. Vertical error bars depict a superconducting transition width. Data for x = 0.34 and 1.0 are extracted from refs 21 and 27, respectively. (**c**) Shielding signals measured under a magnetic field of *H* = 10 Oe.

Low-energy spin fluctuations of nearly optimum hole-doped Ba_1−x_K_x_Fe_2_As_2_ (x = 0.34) exhibit commensurate peaks at Q = (0.5, 0.5, *L*)^21^. These magnetic peaks split along the longitudinal direction forming incommensurate peaks at Q = (0.5 ± δ, 0.5 ± δ, *L*) for x > 0.5 (Fig. 1). Already for the x = 0.50, slightly overdoped sample with a T_c_ of 36 K, double peaks were observed with an overlapping structure. These peaks separate completely as doping further increases, see also^19^,^27^. Our results qualitatively agree with the previous INS study on powder samples^22^ and demonstrate that the incommensurability δ increases with increasing doping level associated with the suppression of *T*_*c*_ from the optimum-doped region. As shown in Fig. 1(b), the suppression of *T*_*c*_ with increasing δ follows a parabolic behavior up to x = 0.66. This clear relationship suggests that the periodicity of the spin fluctuations has a considerable impact on superconductivity and that a commensurate structure is advantageous for achieving high *T*_*c*_. The parabolic correlation, however, ends near x = 0.66, where *T*_*c*_ drops dramatically. This suggests that the interplay between magnetism and superconductivity changes around x = 0.66.

**Figure 2:**
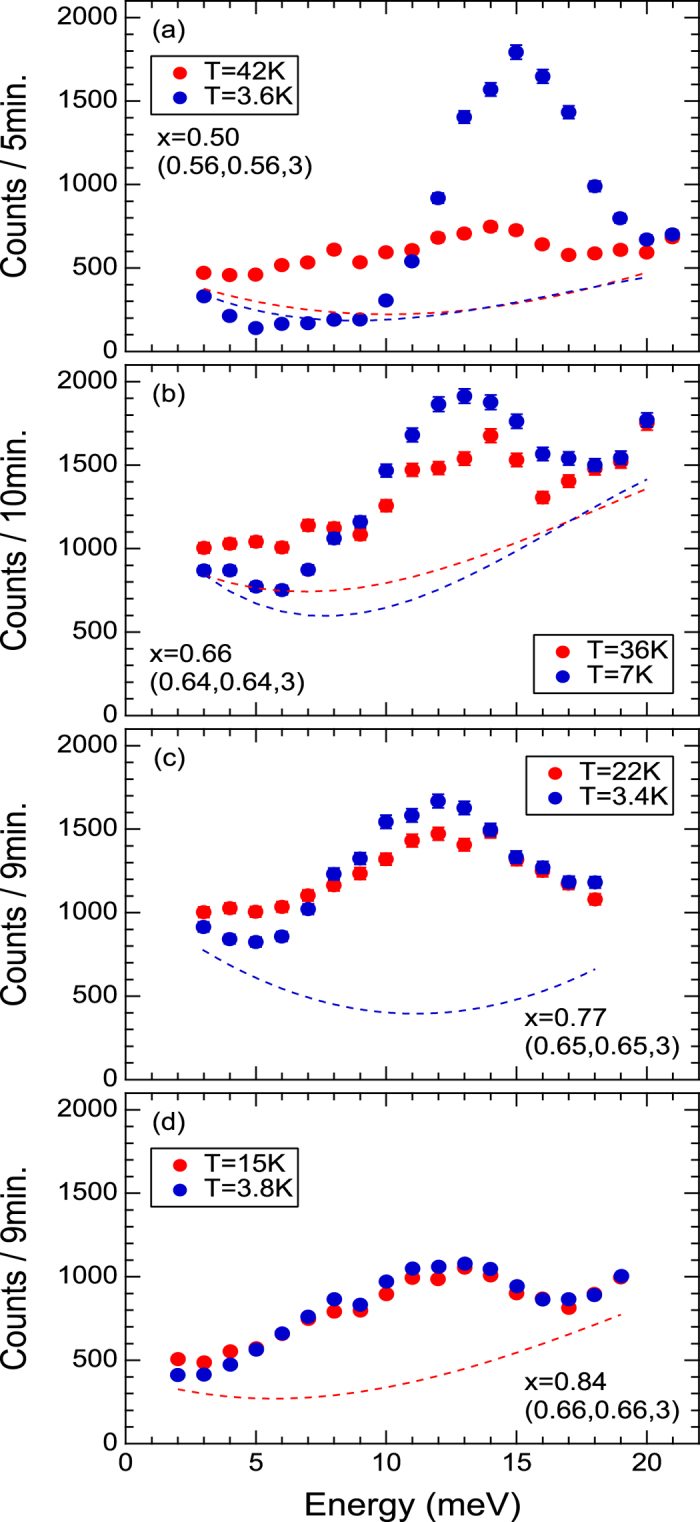
Energy dependences of magnetic signals at Q = (0.5 ± δ, 0.5 ± δ, 3) at T ~ T_c_ and T < T_c_ for (**a**) x = 0.50, (**b**) x = 0.66, (**c**) x = 0.77 and (**d**) x = 0.84. Dashed lines describe the background at T ~ T_c_ (red) and T < T_c_ (blue) determined by averaging the background at (**a**) (0.28, 0.28, 4), (0.72, 0.72, 3) and (0.695, 0.695, 0); (**b**) (0.48, 0.48, 3.65), (0.22, 0.22, 3) and (0.78, 0.78, 3); (**c**) (0.2, 0.2, 4.29) and (**d**) (0.5, 0.5, 3.69). Because *T*_*c*_ is sufficiently low for x ≥ 0.77 samples, their backgrounds should be almost temperature-independent below *T*_*c*_.

Figure 2 and Supplementary Figure S2 show energy dependences of magnetic signals at T ~ *T*_*c*_ and T < *T*_*c*_ (See also Supplementary Fig. S1). Backgrounds were determined at the sides of the magnetic rods. Huge enhancement of signals below T_c_ is observed at E = 15 meV for x = 0.50 samples, which can be identified as a spin resonance. The enhancement weakens with increasing doping, but still exhibits slight enhancement even in x = 0.84.

**Figure 3:**
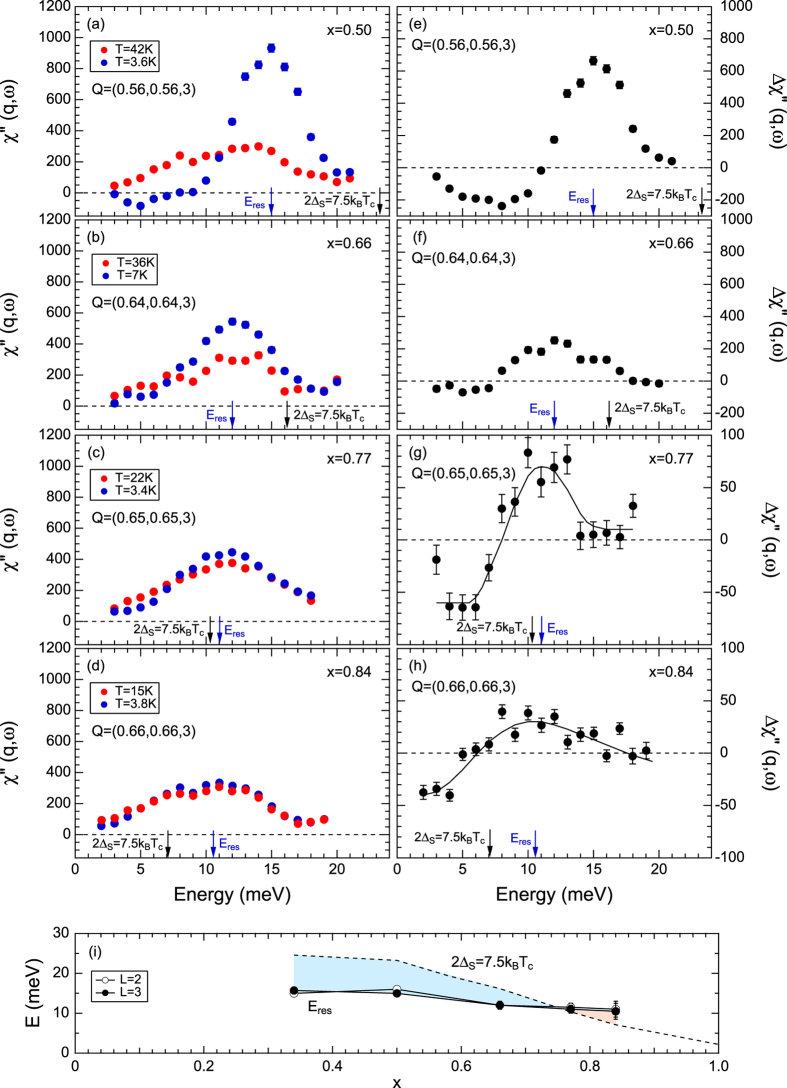
(**a–d**) Energy dependences of χ″(q, ω) at peak positions in Q-scans at T ~ T_c_ and T < T_c_ for (**a**) x = 0.50, (**b**) x = 0.66, (**c**) x = 0.77 and (**d**) x = 0.84. Amplitudes were normalized by phonon scattering intensities. (**e**–**h**) Difference of χ″(q, ω) between T ~ T_c_ and T < T_c_. (i) Doping dependences of *E*_*res*_ at *L* = 2 and 3 with data of x = 0.34 extracted from ref. 21. *E*_*res*_ is almost independent of *L*. The dashed line depicts the superconducting gap value of 2Δ_s_ = 7.5k_B_T_c_.

The imaginary part of dynamical magnetic susceptibility χ″(q, ω) was obtained by multiplying the net intensities by [1 − exp(−*ℏ*ω/*k*_*B*_*T*)] after normalizing them by acoustic phonon intensities and correcting for higher-order components in the incident beam monitor [Fig. 3(a–d); Supplementary Fig. S3(a–d)]. We can obtain χ″(q, ω) in semi-absolute units by this procedure, which allows us to quantitatively compare the magnetic signals within the series of Ba_1−x_K_x_Fe_2_As_2_. χ″(q, ω) at E ≤ 21 meV in the normal state seems to be nearly independent of the hole concentration. Peak structures are observed around E = 12 meV in all samples and the magnitude of χ″(q, ω) is almost constant with doping. The absence of a sizeable variation of the normal-state magnetic response is remarkable in view of the pronounced changes of the Fermi surface topology reported for these compounds. In contrast, χ″(q, ω) in the superconducting phases exhibits a dramatic doping dependence. At x = 0.50, well-defined spin resonance peak is observed at *E*_*res*_ = 15 meV, where χ″(q, ω) increases by a factor of four upon cooling [Fig. 3(a,e); Supplementary Fig. S3(a,e)]. On the other hand, signals below E = 10 meV are drastically suppressed below *T*_*c*_, demonstrating the opening of a spin gap. As the hole doping is further increased, the resonance intensity decreases drastically in qualitative agreement again with the previous study on powders^22^, while *E*_*res*_ stays almost constant (Fig. 3; Supplementary Fig. S3). Finally, only slight differences are observed between χ″(q, ω) at T = *T*_*c*_ and at T ≪ *T*_*c*_ for x = 0.84 that cannot be attributed to a resonance mode. According to ARPES measurements, the value of the maximum 2Δ_s_ is 7.5k_B_T_c_ in the overdoped region^28^, which corresponds to 23.3 meV for x = 0.50. Apparently, the obtained *E*_*res*_ is well below 2Δ_s_ = 7.5k_B_T_c_ for x = 0.50. On the other hand, there is no comparable intensity enhancement below E = 7.5k_B_T_c_ (7.1 meV for x = 0.84) under cooling for x = 0.84; instead, broad and slight enhancement was observed at higher energies near E = 10.5 meV, which is certainly above 7.5k_B_T_c_. There is no evidence that a spin resonance mode appears below 2Δ_s_ for x = 0.84. Assuming that the broad peak in the temperature difference around E = 10.5 meV can be associated with the resonance, *E*_*res*_ exceeds 2Δ_s_ above x = 0.77 [Fig. 3(i)].

**Figure 4:**
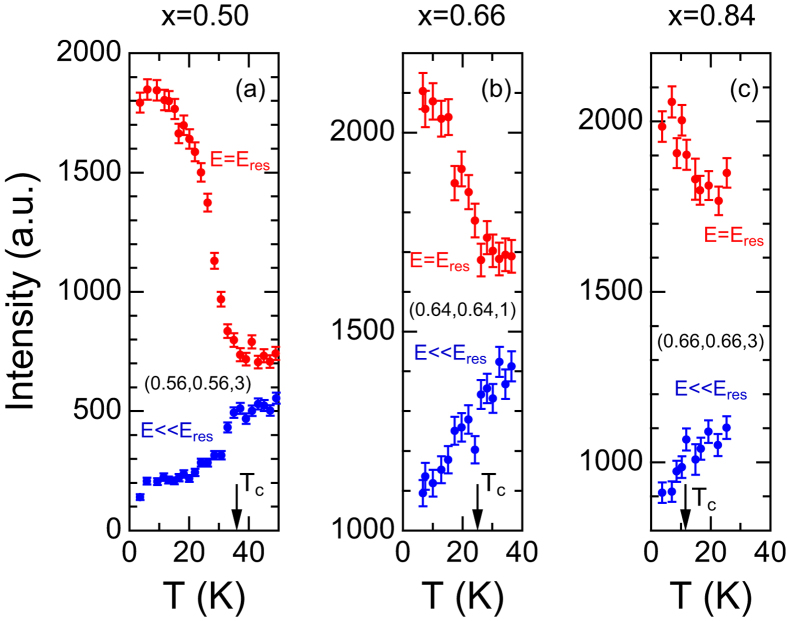
Temperature dependences of intensity at E = *E*_*res*_ and E < Eres. (**a**) Q = (0.56, 0.56, 3), E = 15 meV and 5 meV for x = 0.50, (**b**) Q = (0.64, 0.64, 1), E = 12 meV and 3 meV for x = 0.66 and (**c**) Q = (0.66, 0.66, 3), E = 12 meV and 2 meV for x = 0.84. Intensities at E = *E*_*res*_ increase with decreasing temperature below T_c_, whereas those at E < *E*_*res*_ decrease.

The development of the spin resonances and the gap opening can also be confirmed by the temperature dependence of the scattering intensity. The intensity at E = *E*_*res*_ increases and that at E ≪ *E*_*res*_ decreases below *T*_*c*_ in the overdoped samples including x = 0.84 (Fig. 4). This ensures that the broad and slight intensity enhancement at x = 0.84 can be attributed to the appearance of superconductivity.

**Figure 5:**
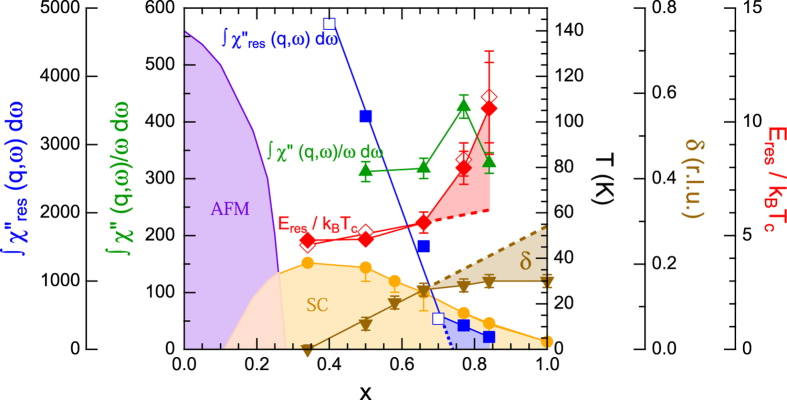
Phase diagram of Ba_1−x_K_x_Fe_2_As_2_. Symbols denote T_c_ (circle), *E*_*res*_/k_B_T_c_ for *L* = even with data of x = 0.34 extracted from ref. 21 (open diamond), *E*_*res*_/k_B_T_c_ for *L* = odd with data of x = 0.34 extracted from ref. 21 (closed diamond), energy-integrated Δχ″(q, ω) around *E*_*res*_ defined as ∫ χ″_res_(q, ω)dω (closed square), ∫ χ″_res_(q, ω)dω extracted from ref. 22 normalized at x = 0.50 (open square), energy integrated χ″(q, ω)/ω from 3 to 18 meV in a normal state (triangle) and incommensurability at T ~ T_c_ (inverted triangle). Dashed lines are extrapolation.

The doping dependences of the obtained magnetic parameters are summarized in Fig. 5. Integrating χ″(q, ω)/ω over energies up to E = 18 meV yields nearly doping independent values, suggesting that the related real part of the dynamical susceptibility defined as χ′(q, ω) = 1/π∫ χ″(q, ω)/ωdω by the Kramers-Kronig relation has no direct relationship with *T*_*c*_. Note that, however, the high-energy response may considerably decrease with the doping as it is suggested by the results for the end member KFe_2_As_2_^29^. The doping dependent incommensurability indicates a clear relationship with *T*_*c*_ up to x = 0.66 [Fig. 1(b)]. In contrast, δ becomes insensitive to the doping level as well as to *T*_*c*_ above x = 0.66, indicating an essential change of the coupling between spin fluctuations and superconductivity. Furthermore, the spin resonance modes vary drastically with doping. The dramatic suppression of the resonance intensities itself can be explained by the spin exciton model, where the resonance intensity is proportional to 2Δ_s_ − *E*_*res*_ under the restriction that *E*_*res*_ should be smaller than 2Δ_s_^30^. In fact, *E*_*res*_ is smaller than 2Δ_s_ and approaches 2Δ_s_ with increasing doping, keeping *E*_*res*_/k_B_*T*_*c*_ almost constant value of 5 below x = 0.66. On the other hand, *E*_*res*_/k_B_*T*_*c*_ increases drastically above x = 0.66 and reaches values of 10.5 at x = 0.84. Such high value can no more be explained by the spin exciton model, which cannot yield a resonance feature above 2Δ_s_ within the continuum of particle-hole excitations. Other mechanisms such as the orbital fluctuation model should be considered.

## Discussion

In overdoped region, there is thus a dramatic change in the magnetic response in the superconducting state, which contrasts with the small degree of variation in the normal state. There can be various reasons for the suppression of the resonance mode. A decrease of the nesting properties can explain such suppression, but this should also have a strong impact on the normal state response. The complex gap structure in overdoped Ba_1−x_K_x_Fe_2_As_2_ can effectively suppress the resonance signal, which arises from averaging particle-hole processes over the entire Fermi surface. Finally, the reduction of the correlation strength indicated by the reduced bandwidth of magnetic excitations in KFe_2_As_2_^29^ may also contribute to the suppression of the resonance; the reduced correlations prohibit a clear separation of the bound resonance state from the continuum of particle-hole excitations.

The intensity enhancement at T < *T*_*c*_ above x = 0.77, on the other hand, cannot be explained by the spin-exciton model. One interpretation is a renormalization of the particle-hole continuum accompanied by the opening of the superconducting gap. This has been discussed in LiFeAs^23^,^31^, where the suppression of the resonance mode resembles the present results in overdoped Ba_1−x_K_x_Fe_2_As_2_. In LiFeAs, the crossover of the spectral weight lies still below the maximum values of 2Δ_s_, like the present x = 0.77 results.

Another possibility is based on the orbital fluctuation model. This leads to the idea that spin fluctuation and orbital fluctuation models compete in iron-based superconductors. The spin fluctuation model can be dominant in the underdoped region, which is close to the three-dimensional AFM phase, as it is suggested by the strong resonance mode well below 2Δ_s_. In fact, the dispersive and anisotropic characters of the spin resonance are observed in the underdoped and optimum doping regions, which can be well explained by the spin-exciton model^16^. In the overdoped region, on the other hand, the three-dimensional character of the AF correlations is completely lost and spin fluctuations appear at an incommensurate vector. Owing to the change of those magnetic circumstances, the relationship between spin fluctuation and superconductivity seems to vary dramatically in the overdoped region. A transition from spin fluctuation to orbital fluctuation or some other model can occur in overdoped region of Ba_1−x_K_x_Fe_2_As_2_.

## Methods

Single crystals of Ba_1−x_K_x_Fe_2_As_2_ were grown by the self-flux method^32^. The obtained single crystals had a tabular shape with (001) planes as their surfaces. They were coaligned on a thin Al sample holder to increase their total volume for inelastic neutron scattering experiments. The total weights of single crystals were 1.32, 0.82, 0.69, 0.85 and 0.64 g for x = 0.50, 0.58, 0.66, 0.77 and 0.84, respectively. Composition was determined by the energy dispersive X-ray analysis as well as lattice constant c examined by x-ray diffraction. Composition distribution among each assembled single crystals was confirmed to be less than ±0.04 in x value by examining lattice constant c of all single crystals using x-ray diffraction on both sides of sample surfaces. Superconducting transition temperatures were examined by SQUID magnetometer (Quantum Design MPMS) under a magnetic field of 10 Oe after zero-field-cooling. Onset temperatures of T_c_ were 36, 30, 25, 16 and 11.5 K with a transition width of 6, 5, 8, 5, 4 K for x = 0.50, 0.58, 0.66, 0.77 and 0.84, respectively. Inelastic neutron scattering measurements were conducted at FRM ll on the triple-axis spectrometer PUMA and at LLB on the 2T1 spectrometer. The final neutron energy was fixed at *E*_*f*_ = 14.7 meV by using double-focusing pyrolytic graphite crystals as a monochromator and analyzer. To remove higher-order neutrons, a pyrolytic graphite filter was inserted between the sample and the analyzer. Data of x = 0.50, 0.58, 0.66 and 0.77 were measured at PUMA and that of x = 0.84 was measured at PUMA and 2T1.

## Additional Information

**How to cite this article**: Lee, C. H. *et al*. Suppression of spin-exciton state in hole overdoped iron-based superconductors. *Sci. Rep.* **6**, 23424; doi: 10.1038/srep23424 (2016).

## Supplementary Material

Supplementary Information
