# Supplementary material for: Suppression of spin-exciton state in hole overdoped iron-based superconductors
Source: Sci Rep. 2016 Mar 23;6:23424. doi: 10.1038/srep23424 (PMC4804212; doi:10.1038/srep23424)
Supplement: Supplementary Information [file srep23424-s1.pdf]

Suppression of spin-exciton state in hole overdoped iron-based superconductors

C. H. Lee[1], K. Kihou[1], J. T. Park[2], K. Horigane[3], K. Fujita[3], F. Waßer[4], N. Qureshi[4], Y. Sidis[5] J. Akimitsu[3], and M. Braden[4]

[1]National Institute of Advanced Industrial Science and Technology (AIST), Tsukuba, Ibaraki 305-8568, Japan. [2] Heinz Maier-Leibnitz Zentrum (MLZ), Technische Universität München, D-85748 Garching, Germany, [3]Aoyama Gakuin University, Sagamihara 252-5258, Japan. [4]University zu Köln, Germany, [5]Laboratoire Léon Brillouin (LLB), C.E.A./C.N.R.S., F-91191 Gif-sur-Yvette Cedex, France. [*]E-mail: c.lee@aist.go.jp

1. Single crystal growth

Single crystals of $Ba_{1-x}K_xFe_2As_2$ were grown by the self-flux method [S1]. The starting materials were Ba (99.9%), K (99.9%), Fe (99.99%), and As (99.9999%). First, we synthesized the precursors KAs, BaAs, Fe_2As and FeAs. Starting materials were put into alumina crucibles encapsulated into stainless steel containers filled with dried N_2. They were then heated for 10 h at 650 ˚C for KAs and BaAs and 900 ˚C for FeAs and Fe_2As. The obtained precursors were mixed and encapsulated into a stainless steel container with an alumina crucible again. The container was heated up to 900 ˚C and then cooled down to 650 ˚C at a rate of 1˚C / h. The single crystals thus obtained had a tabular shape.

2. Results of inelastic neutron scattering

Figure S1 shows the Q-spectra of $Ba_{1-x}K_xFe_2As_2$ (x=0.50, 0.66, 0.77 and 0.84) at E < E_{res} and E ~ E_{res} above and below T_c. A remarkable enhancement of intensity was observed at E ~ E_{res} upon cooling for x = 0.50. The enhancement weakened as doping

increased and became small for x = 0.84. Suppression of intensity by cooling was observed at E < E_{res}. For x = 0.50, magnetic peaks at E = 5 meV vanished below T_c. On the other hand, weak incommensurate intensity at E = 3 or 4 meV remained for x ≥ 0.77, although the intensity was suppressed upon cooling.

Energy dependences of magnetic signals at T ~ T_c and T < T_c with L = 2 are shown in Figure S2. Figure S3 shows $\chi''(q,\omega)$ at Q = (0.5 ± δ, 0.5 ± δ, L = *even*). The behavior of $\chi''(q,\omega)$ at L = even is essentially the same as that at L = odd. The difference in E_{res} between L = even and odd is within 1 meV.

References

S1. K. Kihou, T. Saito, S. Ishida, M. Nakajima, Y. Tomioka, H. Fukazawa, Y. Kohori, T. Ito, S. Uchida, A. Iyo, C. H. Lee and H. Eisaki, J. Phys. Soc. Jpn. **79**, 124713 (2010).

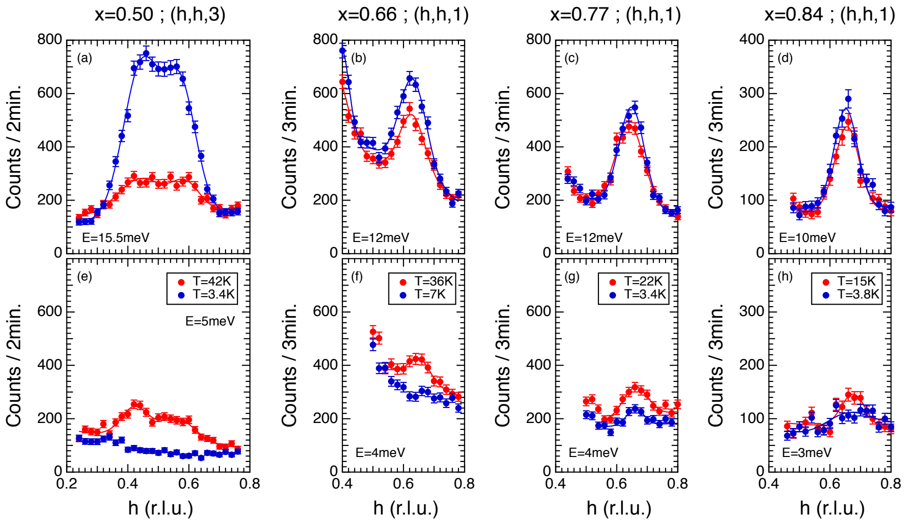

Figure S1. (a) Q-spectra of $Ba_{1-x}K_xFe_2As_2$ at E ~ E_{res} and E < E_{res} at T ~ T_c and T < T_c for (a) x = 0.50, (b) x = 0.66, (c) x = 0.77 and (d) x = 0.84. Solid lines indicate Gaussian fits.

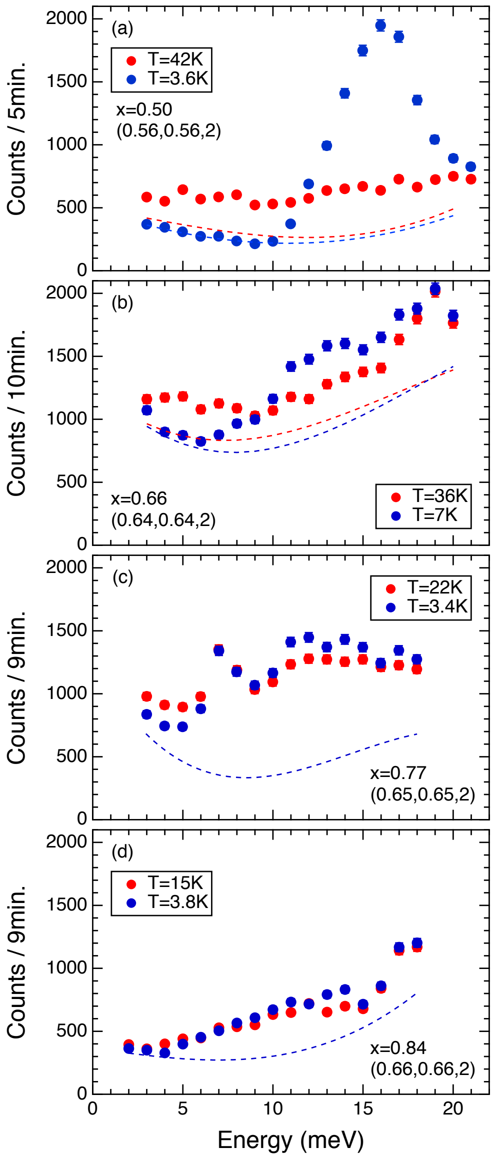

Figure S2. Energy dependences of magnetic signals at Q = (0.5 ± δ, 0.5 ± δ, 2) at T ~ T_c and T < T_c for (a) x = 0.50, (b) x = 0.66, (c) x = 0.77 and (d) x = 0.84. Data for (d) were obtained at 2T1 and others were obtained at PUMA. Dashed lines describe the background at T ~ T_c (red) and T < T_c (blue) determined by averaging the background at (a) (0.28,0.28,3) and (0.695,0.695,0); (b) (0.5,0.5,2), (0.5,0.5,3), (0.22,0.22,2) and (0.78,0.78,2); (c) (0.2,0.2,3.66) and (d) (0.2,0.2,3.74) and (0.5,0.5,2.95).

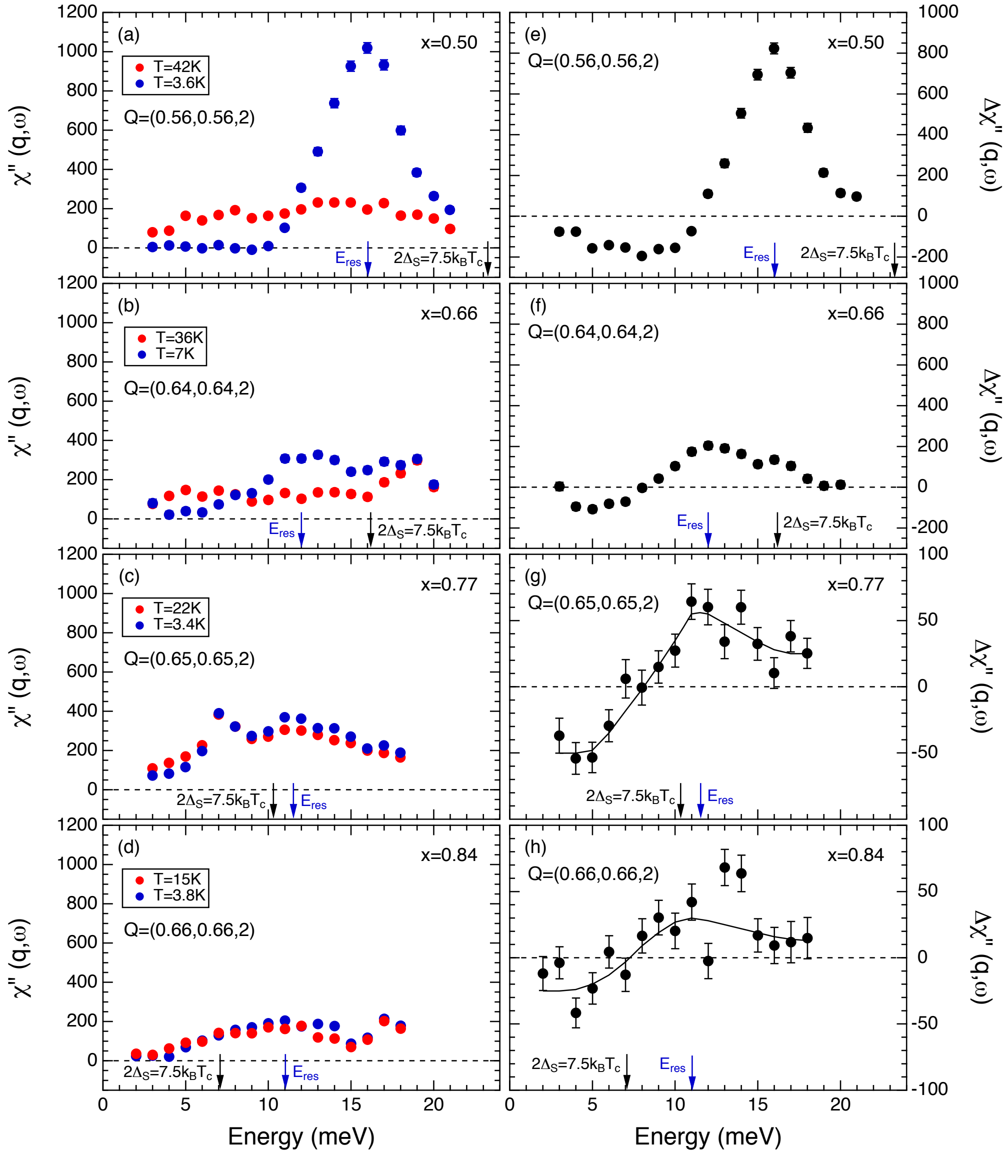

Figure S3. (a-d) Energy dependences of $\chi''(q,\omega)$ at $Q = (0.5 \pm \delta, 0.5 \pm \delta, 2)$ at $T \sim T_c$ and $T < T_c$ for (a) $x = 0.50$, (b) $x = 0.66$, (c) $x = 0.77$ and (d) $x = 0.84$. (e-h) Difference in $\chi''(q,\omega)$ between $T \sim T_c$ and $T < T_c$. The solid lines are a guide to the eye.
